# Reason, Genius, and Madness

**Published:** 1861-01

**Authors:** 


### Art. XI.—REASON, GENIUS, AND MADNESS.

Not long ago a French physician of note, one upon whose opinions medical psychologists had long been accustomed to look with respect, gave to the world a work,* the object of which was to prove that *genius* and *insanity* were so closely allied, that they sprang from the same stock, were twin branches upon the same tree. *Genius*, in short, according to this author, was, as insanity, a morbid affection of the nervous system—"*a neurosis*," as he expresses it; both the one and the other were developed by the action of the same series of causes, and such difference as existed between them was one of degree and not of essential character. This strange paradox was supported by an amount of ingenuity, learning, and actual observation, which can only be rightly appreciated by a reference to the book itself. Nay, so well is the book written, that the reader is almost compelled to exclaim, parodying the scene in the celebrated consultation on *Monsieur de Pourceaugnac*, when the second physician expresses his admiration of the skilful diagnosis and prognosis of the senior physician:—"The arguments you have advanced are so learned and beautiful, that it is impossible that one should be a genius without being a madman; and if it were not so, it ought to be so, from the excellency of the things you have said, and the justness of your reasoning."†

At the time when the work was published, we devoted some attention to it,‡ and endeavoured to show that the theory advocated was but a new version of a very old story; and that when stripped of all extraneous matter, and reduced to a simple question of the signification of terms, the proposition that genius was a neurosis, was no better than a play upon words, such, in reality, as that by which Touchstone routed the straightforward reasoning of the clown, and which may be aptly paralleled by an ingenious gastronomical paradox, which finds a place in the pages of the welcome almanack for the present year of our respected contemporary *Punch*:—"Buck-venison is the best for venison-pasty, although the pasty is made of dough."

If we return to the subject of M. Moreau's work now, it is

---

* *La Psychologie Morbide.* Par Dr. J. Moreau (de Tours). Paris. 1859.

† Act i. Sc. I. sc. xi.

‡ See article "Paradoxical Psychology," *Journal of Psychological Medicine*, vol. xiii. p. 1.

because the book has derived an additional interest from the fact of Professor Flourens, having deemed it worth while to prepare and publish a species of antidote against it in the form of a small octavo volume.

"There are men," wrote Leibnitz, at the commencement of the last century, "who believe that it is witty to declaim against reason. . . . I see little books and trifling disquisitions, in which this notion is made much of, and sometimes even I see verses much too beautiful to be devoted to such false thoughts. In truth, if those who mock reason speak in good faith, this would be a new species of extravagance unknown in past ages." But, now-a-days, remarks Flourens, commenting on this passage, we do not restrain ourselves to mocking reason merely; we go far beyond this. We produce voluminous and learned works to prove that genius is a malady. Let us add, lest it should be imagined that M. Moreau's theory applies only to the exceptional intellectual manifestations usually classed under the term genius, that so far from this being the case, the time-honoured maxim "*mens sana in corpore sano*" of necessity falls to the ground, the theory being accepted. "This maxim," writes the last-named writer, "is no longer true: it is requisite to assert precisely the contrary. In truth, if the normal state of the organism accorded generally with the regular action of the thinking faculty, we should never see in this case, or only exceptionally, the intelligence become elevated, as well in its affective as in its intellectual relations properly so called, above an honest mediocrity. In these conditions, man might be endowed with a sense of justice, with a judgment more or less sure, with a certain degree of imagination; his passions would be moderate; always master of himself, he would practise better than any one the doctrine of self-interest; he would never become a great criminal, but he would never become a great man: he would never be attacked with that *mental malady* called *genius:* in other words, he would never, under any circumstances, be ranked among privileged beings."†

Well may M. Flourens dread the loss of all human dignity if this theory were to be established. The "if," however, in this case is of most portentous dimensions. A paradox which contains its own refutation within the space of the words in which it is expressed, can have little chance of being recognised even transitorily as a scientific truth. Yet for all this it will have charms

---

* *De la Raison, du Génie, et la Folie.* Par P. Flourens, Membre de l'Académie Française, et Secrétaire Perpetuel de l'Académie des Sciences (Institut de France). Paris. 1860.

† *La Psychologie Morbide*, p. 468.

for many; and M. Flourens was right when he thought that the appearance of M. Moreau's work bespoke the necessity for a brief and authoritative recapitulation of the great and undoubted signs by which we recognise reason, genius, and madness. This M. Flourens endeavours to effect in the work we have referred to. There may be vast and imperfectly, or altogether unexplored tracts of country between the different beacon-lights, but he who steadily guides his course by these lights will never be led far astray by a Will-o'-the-wisp, or look long upon dense banks of fog as solid ground, or be deceived by the fantastic delusions of a mirage. He may, for the moment, mistake the flitting fire of the *ignis fatuus* for the blaze of the true beacon; his true course may be hidden in the fog, or, deluded by the mirage, he may think that he sees close at hand and within easy reach the goal at which he aimed; but *if* he be always on his guard, always advance doubtingly when the beacon is hid, and be heedful when the light is in view, there is little fear of his plunging deeply into the slough of mental paradoxes.

"I examine successively in this book," writes M. Flourens of the work before us, "*reason*, the supreme gift of GOD to man; *genius*, the highest expression of that reason; and *madness*, which is nothing else than disorder of our ideas, a disorder which is not *fatal*, and against which the energetic attention of our proper spirit upon itself will always be the most salutary curb."

We do not propose to follow closely M. Flourens' arguments and statements, but simply to dip here and there into his work, selecting one or two illustrations of the manner in which he deals with his subjects.

Terminating his analysis of M. Moreau's work in the section devoted to *Genius*, he writes:—

"In conclusion, I define *genius* a superior reason; and the author defines it as *neurosis*. Definitions are free. But what is the precise fact, what is the characteristic fact of *genius?* It is that it sees, it judges, it approves, it blames, it corrects: this is the certain mark of reason. And what is the precise fact, the characteristic fact of insanity? It is that it cannot either see, or judge, or blame, or correct. These are no longer words, but trenchant facts.

"Socrates and Pascal are continually cited. Socrates believed that he *saw* [heard] a familiar demon; Pascal believed that he *saw* a precipice open under his feet. Both Socrates and Pascal had these hallucinations. But what does this prove? Does it prove that the *hallucination* was genius, or that it produced genius; that without his *hallucination* Socrates would not have possessed his good sense; that without his *hallucination* Pascal would not have had his great mind? Can we not see that all these relations between genius and insanity are but exterior relations, occasional and fortuitous; that they are not *necessary* relations, and that the whole question rests there?

"Every error concerning the nature of things depends upon a fault of analysis. 'I wish that analysis were not bounded,' said Leibnitz. A restricted analysis leads to superficial analogies. A full and entire analysis alone goes to the bottom of things, and sees there the profound distinction which separates *genius*, that supreme power of discerning and seizing the truth from *insanity*, that fatal illusion which gives to the false—that is to say, to that which does not exist—a kind of being." (p. 116.)

Hereditary transmission is the mysterious key with which M. Moreau unlocks the chief difficulties which beset his theory. M. Moreau avers that we are born mad: the primitive and genetic fact of insanity, according to him, is hereditary transmission. Heritage being laid down as a principle, "fatality," M. Flourens states, "follows as a consequence." "When," writes M. Moreau, "in the progenitor of an individual we see the mechanism of innervation diversely modified, injured, and altered, vitiated, indeed, in every way in all its modes of manifestation, functional, dynamic, and organic, it is easy to recognise the kind of pathological fatality of this individual."

"*Hereditary transmission and fatality,*" observes M. Flourens, "are the two dominant points of the whole system." M. Moreau yields but an insignificant place to acquired insanity. On the assumed fatal consequences of hereditary transmission, "or, as we may say, in one word, *the fatality of heredity*" (a coinage convenient to use, as we must have recourse to *inneity*—innéité, hérédité), M. Flourens has some interesting observations which we shall not hesitate to cite, although they may be somewhat lengthy:—

"M. Moreau exhibits *heredity* as a *simple fact;* he deceives himself, it is a *double fact;* a fact *primitive* and a fact *secondary.* Before there were madmen, there were men. Before *heredity*, properly so called, there was *inneity.* Before the immediate parent, there was the general parent, humanity.

"There are in the animal economy, such as we know it at this day, after so many ages of successive generations, two orders of *qualities*—the qualities *innate* and the qualities *acquired*, the qualities *primitive* and the qualities *secondary*, the qualities *essential* and the qualities *accessory*. 'The imprint of each species,' writes Buffon, 'is a type of which the principal traits are graven in ineffaceable and permanent characters for ever, but all the accessory touches vary; no individual perfectly resembles another, no species exists without a great number of varieties. . . .'

"I call those qualities *innate* with which each species has been endowed from its first formation, its *creation*. Dating from its first formation, from its *creation*, the *essential* qualities of each species have remained the same. The lion of to-day is the same as that of the time of Aristotle; the ibis of to-day is the same as that of the time of the Pharaohs. M. Cuvier found the real elephant* more exactly described in Aristotle than in Buffon; no species has changed; the *type* of each persists, and remains immutable. More, when some of the *accessory* qualities have been modified, either by the influence of climate, of food, or man, more powerful to that end than all the other causes together, it is but needed that the extraneous cause should cease to act, and the acquired modifications disappear, and the original character return.

"A decisive experiment to this effect has been made upon a grand scale. After the conquest of the New World, the Spaniards carried there their domestic animals, the horse, the dog, the pig, the ox, the goat, &c. These animals having been restored to their primitive state —that is to say, to freedom—have lost all their characters of *race*, and have retaken their characters of *species*.

"The horse has returned to his natural size, which is about that of the ass; his original colour, which is dark bay; his instinct to live in troops commanded by a chief, &c. The dog has returned to his proper size, that of the jackal; his instinct to burrow, to pursue game in concert, and his ears have become straight; he has also returned to his howling, and ceased to bark, which was the fruit of his relations with man, &c.; the hog has become there a wild boar, and the porker is again clad in his primitive livery.

"The *innate* qualities have then, as compared with the *acquired*, this advantage, that they are permanent and fixed; that hidden on the surface, they survive in the deeper beds, always ready to rise up anew and retake the empire, when the *acquired* qualities no longer find in exterior circumstances the borrowed aids which were requisite to maintain and reproduce them.

"It is of organs as of faculties. We cannot cause the loss of an organ. I removed from hares, dogs, mongrels of the jackal and dog, field mice, the supra-renal capsules, the tail, the ears, the spleen, the thyroid body, and this during four, five, and six generations successively. At each new generation, the organ has been unquestionably removed from each couple—from the male and female, from the father and mother, and the progeny have always re-produced supra-renal capsules, tails, ears, spleens, and thyroid bodies.

"The scholastic axiom mooted with so much satisfaction by our author, 'that no one can give that which he does not possess,' does not hold good here. The father and mother without spleen have, notwithstanding, given their young ones that organ; the tailless father and mother have given their young ones a tail; and the earless father and mother have given their progeny ears.† What a phenomenon! what a mystery! And what proof could be more certain of

---

* The *Asiatic elephant*, the only one known to Aristotle.

† In physiology, the term *congenital* variation is applied to the changes produced spontaneously, which arise from birth: these alone can be transmitted, and form a *race*. *Accidental* variations (*mutilations*) are never hereditary.

this primitive and ever-subsisting power, which tends to bring back without ceasing the things of *primary institution*, of *creation*, and to correct and eliminate vicious things, changed by subsequent generations?

"This power of nature, of *recall* (if I may so phrase it), to things of *institution*, of *formation*, of *origin*, is the first resource of the organism against the *fatality of heredity*.

"And there is still another matter arising even from *heredity* itself.

"M. Moreau insists much, and rightly, upon 'the influence [it is he who speaks] of marriages effected against the laws of a healthy physiology.' And here what a path he opens to delicate and judicious medications!

"Nothing is better known than the art of producing, among our domestic animals, smaller or greater races. By uniting together at each generation, smaller and still smaller individuals, in the end are produced those little pet-dogs, 'which,' remarks M. Cuvier, 'are the most degraded products, and the strongest indication of the power that man exercises over nature.'

"I have begun an inverse series of experiments. I propose to obtain the greatest individuals that the two species of the wolf and the dog, united together, can give rise to. At each generation the greatest males are united with the greatest females. At the end of three generations, enormous and extremely ferocious animals were obtained.

"I say *extremely ferocious;* and this is a trait to be noted here, because that which is bad in the organization is transmitted and increased by assorted combinations, as well as that which is good.

"Bad races may be engendered as well as good; the bad, indeed, are more quickly obtained than the good . . . . . *Heredity* then causes, by turns, good and evil. It ameliorates, it deteriorates, it vitiates, it perfects, according as the series of generations is well or ill-conducted; the whole secret is in the art of *assorted combinations*.

"But, putting aside this art, all *human*, of *assorted combinations*, and looking to nature alone, we see that, at the bottom, the species remains always the same. 'The species will be always new,' Buffon has said. 'To the eyes of the man who judges grandly and generally,' he again says, 'the one thousandth animal in the order of generations is the same as the first.' And he confirms, enlarges, and illumines his reasoning by a beautiful image. He places, by a philosophical fiction, the *species* in the place of the *individual*.

"'Let us place,' he writes, 'the species in the place of the individual; we have seen what the spectacle of nature was for man; imagine we what the view would be for a being who represents the entire human species. . . . . Man, he continues, coming into the world, comes into darkness: the soul as naked as the body, he is born without knowledge and without defence; he brings with him only passive qualities; he can but receive the impressions of objects, and suffer his organs to be affected; the light burns long before his eyes before enlightening them: at first he receives everything from nature, and returns nothing to it; but when his senses are strengthened, when he can compare his sensations, he throws himself back towards the universe; he forms ideas, preserves them, comprehends them, combines them. Man, and especially instructed man, is no more a simple individual; he represents in great part the entire human species; he has commenced by receiving from his fathers the knowledge which had been transmitted to them by their ancestors; these, having discovered the divine art of tracing the thoughts and of giving them to posterity, are, so to speak, identified with their nephews, ours identify themselves with us; this re-union, in a single man, of the experience of many ages, removes to infinity the limits of his being; he is no more a simple individual, bounded, as others, to the sensations of the present moment, to the experiences of the actual day: he is almost the being that we have placed in the position of the entire species; he reads in the past, sees the present, judges the future, and in the torrent of time, which reduces, carries away, swallows up all the individuals in the universe, he finds the species constant, nature invariable: the relation of things being always the same, the order of time appears to him nothing; the laws of renewal, in his eyes, only compensate those of permanence; a continual succession of beings, all similar the one to the other, is equivalent in effect to the perpetual existence of one of these beings alone.'

"From this brilliant fiction let us return to facts. The novelty of the species is then eternal. And how does this come to pass? Because each new generation is as a new effort towards the *restitution*, towards the *reparation* of the species; because there are two orders of qualities, as I have already said, the *essential* and the *accessory*, the *innate* and the *acquired*, the *primitive* and *healthy*, the *secondary* and *vitiated*, and the inherent force of the organism tends without ceasing to reascend from the one to the other: from the *acquired* to the *innate* qualities, from the *accessory* to the *essential*, from the *secondary* to the *primitive*, and from the *vitiated* to the *healthy*.

"Thus much for *heredity*.

"*Heredity*, explained, leaves no place for *fatality*. *Destiny* no more exists than *chance*. *Destiny* and *chance* are two words which have never indicated more than one thing: ignorance of causes. 'Destiny neglects causes,' remarks Liebnitz; and, long before him, the poet Virgil, that other great philosopher, had said:—

> "'Felix qui potuit rerum cognoscere causas,
> Atque metus omnes et inexorabile fatum
> Subjecit pedibus . . . . . . .'" (p. 12.)

If this quotation be lengthy, we would claim for it, apart from its intrinsic merits, the advantage of being an excellent example of the method in which M. Flourens treats the subject-matters of his book. Hereditary transmissions has of late, moreover, been a subject of so much familiar as well as scientific gossip (if we may so speak without offence), that the popularized opinion of the distinguished Perpetual Secretary of the

Academy of Sciences on the question possesses an interest altogether foreign from the immediate object of the writer.

We shall simply refer to one subject more in M. Flourens' work. "And, now," he asks, when summing up the section of the book devoted to *Genius*, "what is genius?" This is the reply:—

"*It is the power, carried to the highest degree, of thinking justly and of seizing the truth.*

"*In every mode of life there are routes which lead to the truth.*

"*The man of genius is he who opens these routes.*" (p. 165.)

This admirable view of genius would alone checkmate M. Moreau.

M. Flourens' work is throughout most pleasant reading, and will undoubtedly be most widely read. The antidote, indeed, if we err not, will be made use of from its very gratefulness, where the bane has never reached. On those points of the book, chiefly secondary, which we think are open to criticism, we shall offer no remark. We have read the book with reference to its main object, and would recommend others to do the same.

The more we reflect on M. Moreau's theory, the more untenable does it appear to us. M. Moreau must pardon us, but we know no parallel to it except it may be (in kind, at least,) the opinions of the worthy physicians who visited M. de Pourceaugnac. The unhappy man of genius, or individual whose intellect chances to be raised a little above a stagnant mediocrity, holds much the same position towards M. Moreau that the unfortunate gentleman in Molière's comedy did towards the two physicians. M. Moreau's theory has a most insatiable maw.

"The least eccentricity," truly writes M. Flourens, "the most insignificant trait of apparent foolishness or distraction, the most simple emotion, and, if I may say so, the most reasonable, a nervous disorder, whatever it may be, the least suspicion of rachitis, all these things are accounted as so many accusing proofs of a manifest *consanguinity* with insanity."

Thus it happens that there figure upon the author's (M. Moreau's) list [of facts illustrative of his theory]:—Newton, because he was seized with despair at the sight of his burning manuscripts; a despair very natural when we reflect on the character of those manuscripts: Malherbe, because he had a *very disagreeable vice of pronunciation;* Leibnitz, "because his niece," (it is the author who writes,) "who was his heir, having found, after the death of her uncle, sixty thousand ducats in a box under the bed, died on perceiving them, not imagining, says Zimmerman, that a philosopher would possess money." "Turenne, because he *stammered*, and shrugged his shoulders from time to time while speaking." Bossuet, because his head ("that head so vigorous," as the author well says,) was all at once disturbed, when he was given to understand that he must undergo the operation for stone. Montesquieu, because towards the end of his life he *was struck with blindness;* Cuvier, *because he died from an affection of the nervous centres;* Talleyrand, because he had *club-foot;* Napoleon, because he was *round-backed.* "A painter, who had very often the opportunity of seeing and observing the emperor, has remarked to me that he had an excessively arched back, or, as we vulgarly say, he was round-backed (*le dos rond*)." (p. 114)

We read in Molière how, after the victimized but sane M. Pourceaugnac had been pronounced to be mad by the physicians, everything that he did or said was set down by them as a further proof of his madness. After the unfortunate gentleman had submitted long and patiently to the flood of technicalities, in which the learned doctors expounded their opinions of his intellectual state, he exclaimed (Act i. Scene xi.):

*M. de Pourceaugnac.*—Gentlemen, I have listened to you for an hour: are we acting a Comedy?

*First Physician.*—No, sir, we are not acting.

*M. de Pourceaugnac.*—What is all this? What do you mean say with your galimatias and fooleries?

*First Physician.*—Good! he becomes abusive! This confirms our diagnosis: the malady may pass into mania.

*M. de Pourceaugnac.*—With whom am I placed here? (*He spits two or three times.*)

*First Physician.*—Another diagnostic: frequent sputations.

*M. de Pourceaugnac.*—No more of this; let us leave the place.

*First Physician.*—Another still: restlessness.

*M. de Pourceaugnac.*—What does all this mean? and what do you wish?

*First Physician.*—To cure you, according to the order given to us.

*M. de Pourceaugnac.*—To cure me?

*First Physician.*—Yes.

*M. de Pourceaugnac.*—Parbleu! I have nothing the matter with me.

*First Physician.*—A bad sign when a patient does not feel himself ill.

*M. de Pourceaugnac.*—I can tell you that I am quite well.

*First Physician.*—We know better than you how you are; and we are physicians who understand perfectly your constitution.

*M. de Pourceaugnac.*—If you are physicians, I have done with you; I laugh at physic.

*First Physician.*—Oh! oh! this man is madder than we thought.

We feel as if we were exercising a species of righteous retribution in turning a portion of Molière's bitter satire upon M. Moreau's theory, as Molière's name appears among the list of those men of genius who are cited by M. Moreau as illustrations of the truth of his theory. Molière "suffered from convulsions," writes

M. Moreau. "The least check, the least disorder, *brought on convulsions*, and prevented him from working for fifteen days."

We have not the moral courage to resist the quotation, as a tail-piece to this article, of the song of the two physicians in Act I. Scene xiii. of Monsieur de Pourceaugnac, and which almost immediately follows the scene from which we have just quoted. In this song the doctors prescribe the moral treatment of their patient. We chance to possess a very clever and close translation of the song by the learned translator of the latest English version of Dante,* the Rev. J. W. Thomas.

Of the two chief means to be employed in the intellectual treatment of the insane, "the second," writes M. Flourens, following Lauret, "is to occupy the mind vigorously and assiduously with ideas entirely opposed to those which torment the lunatic." Now, M. de Pourceaugnac was pronounced to be suffering from hypochondriacal melancholy; what, therefore, could more fittingly fulfil M. Leuret's second method "of turning, at any cost, the insane from his mad ideas" than the prescript following?—

LES DEUX MÉDECINS

Buon dì, buon dì, buon dì,
Non vi lasciate uccidere
Dal dolor malinconico,
Noi vi faremo ridere
Col nostro canto armonico;
Sol per guarirvi
Siamo venuti quì
Buon dì, buon dì, buon dì.

PREMIER MÉDECIN.

Altro non è la pazzia,
Che malinconia.
Il malato
Non è disparato,
Se nol pigliar un poco d'allegria,
Altro non è la pazzia
Che malinconia.

SECOND MÉDECIN.

Sù, cantate, ballate, ridete;
E, se far meglio volete,
Quando sentite il deliro vicino,
Pigliate del vino,
E qualche volta un poco di tabac.
Allegramente, monsu Pourceaugnac.

---

* Or rather, we should say, of the *Inferno*, the translations of the remaining portions of the Divine Comedy not having yet appeared; but the *Purgatory* is, we believe, announced for publication. Mr. Thomas's translation of the *Inferno* must rank, at least, as one of the two best translations in the English language, and it has the rare merit of being rendered in the triple rhyme of the original.

THE TWO PHYSICIANS.

Good day, good day, good day!
With melancholy grief
Yourselves no longer slay,
But laugh and find relief
In our harmonious lay.
We come but to cure you,
And health to ensure you.
Good day, good day, good day.

FIRST PHYSICIAN.

'Tis madness to give place
To melancholy:
In the worst case
Despair is folly
If you'll but take heart and be a little jolly,
'Tis madness to give place
To melancholy.

SECOND PHYSICIAN.

Up then, and sing, be laughing and dancing:
Still better health if you design,
Whenever the mad fit you feel advancing,
Just take a glass of wine;
With pipe, too, sometimes you may puff away:
So pr'ythee, Monsieur Pourceaugnac, be gay.

